# The Dawning of a New Enterprise: RNA Therapeutics for the Skin

**DOI:** 10.29245/2767-5092/2023/1.1168

**Published:** 2023-03-09

**Authors:** Rachel E. Kieser, Shaheerah Khan, Nada Bejar, Daniel L. Kiss

**Affiliations:** 1 Center for RNA Therapeutics,; 2 Department of Cardiovascular Sciences,; 3 Weill Cornell Medical College, New York, NY, USA; 4 Houston Methodist Cancer Center, Houston, TX, USA; 5 Houston Methodist Academic Institute, Houston Methodist Research Institute, 6670 Bertner Ave, R10-113, Houston 77030, TX, USA

**Keywords:** RNA therapeutics, Skin, mRNA therapeutics, siRNA therapeutics, Antisense oligonucleotide therapeutics, ASO, Melanoma, Hypertrophic scars, Wound healing, Dermatology

## Abstract

Despite being under development for decades, RNA therapeutics have only recently emerged as viable drug platforms. The COVID-19 mRNA vaccines have demonstrated the promise and power of the platform technology. In response, novel RNA drugs are entering clinical trials at an accelerating rate. As the skin is the largest and most accessible organ, it has always been a preferred target for drug discovery. This holds true for RNA therapies as well, and multiple candidate RNA-based drugs are currently in development for an array of skin conditions. In this mini review, we catalog the RNA therapies currently in clinical trials for different dermatological diseases. We summarize the main types of RNA-related drugs and use examples of drugs currently in development to illustrate their key mechanism of action.

## Introduction

The discovery and development of novel RNA therapy candidates has accelerated since the success of the COVID-19 mRNA vaccines ^1^. RNA therapeutics have been tapped to provide novel approaches to treat various dermatological conditions where conventional treatments have offered little success. For example, diabetic patients suffer from poor wound healing, which correlates with an increased chance of infections leading to amputation. In addition, metastatic melanoma is one of the deadliest forms of skin cancer with a 5-year survival rate of less than 5% ^2^. It is inherently resistant to radiotherapy and chemotherapy with median survival reduced to approximately 10 months ^2^. Novel therapies are vital to treat these patients, with several candidate RNA therapies showing promising results.

**Figure 1:**
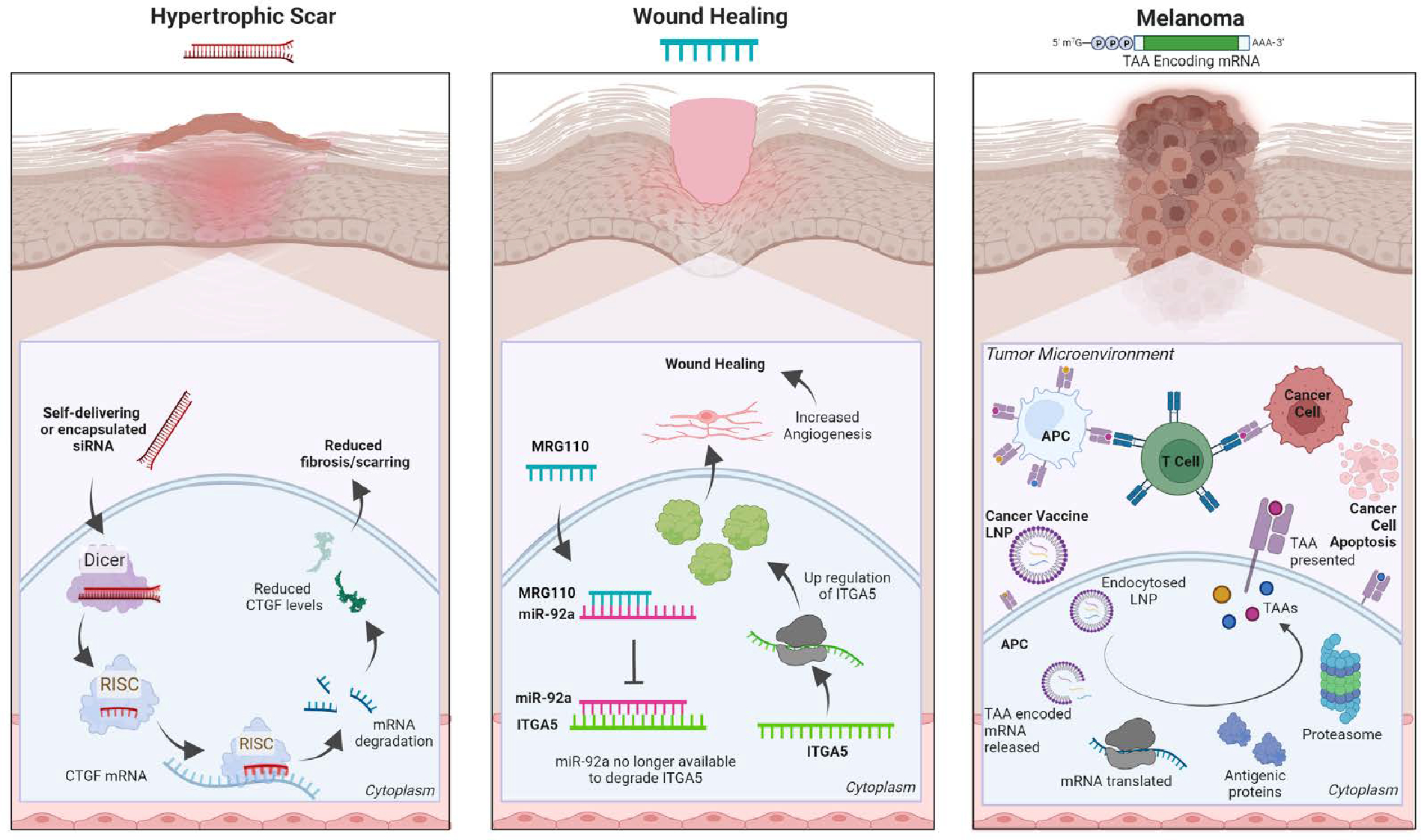
The mechanism of action for three RNA-based therapeutics. (left) Schematic depiction of the mechanism of action for multiple siRNAs currently in clinical trials for hypertrophic scars. All three siRNAs aim to reduce fibrosis and scarring by targeting CTGF mRNA to ultimately lower CTGF protein expression. (Center) The wound-healing candidate drug ASO MRG-110 directly targets miR-92a which represses ITGA5 a pro-angiogenic factor. Blocking the actions of miR-92a increases ITGA5 mRNA and protein levels to promote angiogenesis and wound healing. (Right) The mechanism of action depicting a mRNA-based cancer vaccine (e.g., BNT111) where a pool of mRNA encoding tumor-associated antigens (TAAs), linked to a specific cancer (i.e., BNT111 encodes 4 melanoma antigens), is delivered to antigen-presenting cells (APC) via lipid nanoparticles (LNP). The mRNA pool is translated into TAAs and elicits a specific host anti-tumoral immune response against the cancer cells. Created with BioRender.com.

The rapid advancement of RNA-based therapies underlines their inherent advantages. For example, (1) they have a transient effect, (2) present no risk of insertional mutagenesis, (3) are easy to develop and manufacture, and (4) are cost-effective. These traits make them attractive options for development. Here we summarize the RNA-based therapies currently in clinical trials for skin conditions (Table 1), described are the main categories of RNA therapeutics: RNAi (siRNA and miRNA), antisense oligonucleotides (ASO), messenger RNA (mRNA), and provide examples detailing their mechanisms of action (Figure 1).

## RNA Interference (RNAi)

The aberrant expression of a protein, a mutated protein, or a non-coding RNA ^3,4^ can all cause skin disorders, and blocking such elements can be a beneficial therapeutic approach. RNA interference (RNAi) is one method to specifically target and lower the expression of pathological proteins. In RNAi, short double-stranded RNAs of exogenous or endogenous origins are processed by DICER and loaded into the RNA-induced silencing complex (RISC). Loaded RISC complexes recognize targeted mRNAs via perfect (siRNAs) or imperfect (miRNAs) complementarity and inhibit their translation and/or cause their degradation, thereby reducing the level of the encoded protein ^5–7^. siRNAs are designed to specifically target a single mRNA, whereas miRNAs (naturally occurring or synthetic) often have a broader array of targets ^7^.

The discovery of RNAi has greatly contributed to our understanding of the role(s) of countless proteins and their potential pathogenicity. More importantly to clinicians, RNAi provided a pathway to target disease-causing proteins that were otherwise impossible to treat. As with other RNA platforms, RNA stability, poor tissue penetration, and off-tissue targeting remain challenges; however, modifications to RNA bases and/or backbones plus the development of a wide variety of delivery systems enabled the FDA-approval of multiple siRNA therapeutics ^8^. Although several siRNA and miRNA mimics are currently advancing in clinical trials, no RNAi therapies have yet been approved for skin-related diseases.

Currently, at least three siRNAs (BMT101 (OLX101A), LEMS401, and RXI-109) targeting the connective tissue growth factor (CTGF) are in clinical trials (Figure 1, left). High levels of CTGF have been linked to fibrotic disorders, and reducing CTGF levels is thought to be a viable approach for reducing cutaneous fibrosis ^8–11^. Further, CTGF is also an attractive target for treating keloids or hypertrophic scars, which occur in 40–70% of patients following surgery ^12^.

## Antisense Oligonucleotides (ASOs)

Oligonucleotides are short chains of polymerized nucleotides, and antisense oligonucleotides (ASO) are short (generally 12–30 nucleotides) single-stranded synthetic nucleic acids whose reverse complement sequence allows for the targeting of specific RNA or DNA sequences ^13–16^. The use of oligonucleotides in clinical trials dates back to the late 1950s and early 1960s when methods to synthesize them were first established ^17^. Although tested for decades, Fomivirsen, the first-in-class ASO, was only approved by the FDA to treat cytomegalovirus-induced retinitis in 1998 ^18^.

ASOs can reduce or restore protein expression, inhibit 5’ cap formation, or alter the splicing of targeted mRNAs. ASOs commonly enter the cell via endocytic pathways and recognize their targeted mRNAs through Watson-Crick base pairing ^19,20^. ASOs predominantly function via two mechanisms. First, ASOs can employ an occupancy-mediated degradation mechanism by inducing the cell’s RNase H nuclease activity to degrade a targeted mRNA ^21^. Second, ASOs can use an occupancy-only model and function via steric hindrance. This strategy can up or down-regulate target transcripts by altering their splicing patterns or by masking protein docking sites ^22^.

ASOs are usually trafficked to late endosomes and lysosomes which accounts for their slow release ^23^. Notably, the phosphodiester backbones of unmodified ASOs are prone to endonuclease degradation resulting in comparatively short half-lives; however, chemical modifications can overcome these shortcomings ^24,25^. ASOs are grouped into three generations based on their chemical modifications. (1) First-generation ASOs often replace a non-bridging oxygen atom in the phosphate group with either an amine (phosphoramidites), a methyl group (methyl phosphonates), or a sulfate group (phosphorothioates). When compared to phosphodiester oligonucleotides or unmodified ASOs, first-generation ASOs can resist nucleases and have longer half-lives in plasma ^24^. However, mRNA targeting affinity is slightly reduced due to the decreased melting temperature of these ASOs ^15^. (2) Second-generation ASOs usually have alkyl modifications at the 2’ position of the ribose which improves binding affinity, tissue uptake, and nuclease resistance, while leading to both longer *in vivo* half-lives and lower toxicity ^26^. (3) Third-generation ASOs use chemical modifications to increase their stability, nuclease resistance, and hybridization affinity to the targeted RNA. The most commonly used third-generation ASOs usually incorporate peptide nucleic acids (PNA), locked nucleic acids (LNA), and morpholino phosphoramidite (MF) modifications ^27^. Notably, many third-generation ASOs function by causing steric hindrance of ribosomal machinery or altering the splicing of its targeted RNA ^28,29^.

MRG-110 (Figure 1, center), a third generation LNA ASO developed by miRagen Therapeutics, Inc. (Boulder, CO), is currently in phase 1 clinical trial for wound healing. It blocks miR-92a, which then de-represses the integrin alpha 5 (ITGA5) gene ^30^. Higher levels of ITGA5 protein promotes angiogenesis which facilitates wound healing ^31^. Studies have shown significant results for MRG-110 reporting none or very low systemic toxicity and drug accumulation in distal tissues.

## Messenger RNA (mRNA)

mRNAs are transient RNAs that encode a protein. Exogenous mRNAs were first used to elicit specific protein expression *in vivo* over three decades ago ^32^. Despite that initial success, nearly two decades passed until data were reported for the first clinical trial employing mRNA as a therapeutic ^33^. The development of mRNA-based therapeutics has seen a renaissance as the COVID-19 global pandemic demonstrated their versatility and power. The mRNA-based therapies currently undergoing clinical trials for dermatologically related diseases are listed in Table 1. Three main mRNA treatment modalities have emerged. First, cancer vaccines use mRNAs encoding tumor-specific antigens to stimulate a protective immune response ^1,34^. Second, replacement therapies use mRNAs to produce therapeutic proteins or to counteract the phenotypes of a defective gene/protein. Third, cell-based therapies use mRNA transfected into cells *ex vivo,* with these cells being re-introduced into the patient to modify a specific diseased phenotype/function ^1,35^. Despite the broad applicability of mRNAs, the constraints of this mini review restrict our discussion to mRNA cancer vaccines.

Therapeutic cancer vaccines generally encode tumor-associated antigens (TAAs), or unique markers expressed in cancerous, but not normal, cells. Targeting several TAAs in a single vaccine reduces the risk of tumor antigen escape as it triggers a broad immune response and it aids in the detection of poorly expressed antigens, thereby increasing the robustness of the vaccine and its antitumor response. BioNTech initially pursued a cancer vaccine targeting four melanoma-associated antigens in their phase 1 Lipo-MERIT monotherapy trial ^36^. While this clinical trial is still ongoing, BioNTech has used the Lipo-MERIT data to steer the development of BioNTech’s FixVac (BNT111) therapy for melanoma, which shows a promising safety profile and anti-tumor immune response ^37^. BioNTech’s FixVac mRNA therapeutic platform is a fixed set of mRNA-encoded TAAs known to be expressed in particular cancer types (e.g., melanoma), therefore, prompting a strong and precise immune response against the particular cancer (Figure 1, right). BNT111 was developed to treat patients with anti-PD-1-refractory/relapsed unresectable stage III or IV melanoma and is one of the most promising cancer immunotherapies in development ^38–42^.

Cancerous cells are characterized by their rapid proliferation and expansion which often generates somatic mutations, each of which becomes a potentially targetable neoantigen. A patient’s specific tumor neoantigen profile (mutanome) could be analyzed and incorporated into personalized neoantigen-encoding mRNA vaccines. As neoantigen expression is restricted to tumor cells, this feature could easily be exploited to treat melanomas as they have a high mutation burden ^43^. Recently, Moderna Inc. and Merck & Co. released promising new data regarding their mRNA-based personalized (neoantigen) cancer vaccine, mRNA-4157/V940, for the treatment of melanoma. When the vaccine is administered in concert with Keytruda, a cancer immunotherapy, reports show a 44% reduction in the risk of recurrence or patient death when compared to Keytruda alone over a period of 1 year ^39,43,44^. These data are a major breakthrough in the field of RNA therapeutics, demonstrating the power of mRNA to “train” a patient’s immune system to recognize and attack their specific tumor mutanome, hopefully, translating into durable remission.

mRNA cancer vaccines have many advantages. First, mRNA-based modalities elicit a potent yet reliable immune response to selected antigens *in vivo* and are customizable to a patient’s particular neoantigen profile. Second, mRNA therapies are safe compared to viral vector vaccines as they do not incorporate into the host genome, thus avoiding the risk of insertional mutagenesis. Lastly, mRNA vaccines are relatively inexpensive and rapid to synthesize ^34^. Altogether, mRNA therapeutics hold great promise for the treatment of various dermatological diseases.

## Summary

As mentioned above, RNA therapeutics are a rapidly evolving class of therapies that have the potential to change the face of personalized medicine and revolutionize healthcare^1^. A number of RNA therapeutics have been approved by FDA and many more are in clinical trials for a broad array of indications. In this review, we focused exclusively on RNA therapeutics in clinical trials for dermatological disorders, such as those used to treat melanoma, psoriasis, hypertrophic scars, wound healing, alopecia, epidermolysis bullosa, keloids, etc. Our research uncovered 35 different RNA therapeutics currently undergoing clinical trials and dozens more are in the discovery or preclinical stages of development for skin conditions as well. In closing, many RNA therapies are rapidly progressing through clinical trials and this evolving and growing class of drug candidates offers much promise to improve existing treatment regimens or to become standalone treatments themselves.

## Figures and Tables

**Table 1:** RNA-based therapies currently in clinical trials for skin conditions

Drug name	Trial Phase	Clinical trial #	Status (Jan, 2023)	Skin Condition	Company	Mechanism of action	Alternative names of drug [38, 39]
**RNA interference (RNAi)**							
LEM S401	Phase I	NCT04707131	Active	Hypertrophic Scar & Keloid	Lemonex	Encapsulated siRNA targeting CTGF to reduce fibrosis	LEM-S401; siRNA encapsulated in DegradaBALL
PH 762	Phase I	CSET3432	Active	Malignant melanoma & Squamous cell cancer	Phio Pharmaceuticals	Self-delivering siRNA reducing the expression of PD-1 to enhance T-cells anti-tumor activity (also tested as an Adoptive Cell Therapy)	PH 762 adoptive cell therapy; PH-762; PH762-ACT; RXI 762-ACT
TD101	Phase I	NCT00716014	Completed	Pachyonychia Congenita	Transderm/International Pachyonychia	siRNA targeting one disease-causing mutation of keratin 6a (K6a), the N171K mutant	Pachyonychia congenita siRNA; Reveker; sdTD-K6a.513a.12; siRNA sdTD101; TD-K6a.513a.12; TD101
MRX 34	Phase I/II	NCT02862145/NCT01829971	Terminated	Melanoma	Mirna therapeutics (Synlogic)	Encapsulated synthetic miR-34a mimic- Tumour suppressor gene modulators	miR-34 - Synlogic; miR-34 mimic - Synlogic; MRX-01; MRX34
BMT101 or OLX101A	Phase II	NCT04012099/NCT04877756	Active	Hypertrophic scars	Olix/Hugel	Cell penetrating asymmetric siRNA targeting human CTGF to reduce fiborsis	asiRNA therapeutics - OliX Pharmaceuticals; BMT 101; cp-asiRNA therapeutics - OliX Pharmaceuticals; OLX 10020; OLX 103; OLX 201; OLX 301; OLX 401; OLX 701;OLX10010; OLX101
STP 705	Phase II	NCT04844840/NCT05196373/NCT04844983/NCT05421013	Active	Squamous Cell Carcinoma, Keloids & hypertrophic scar, Fat sculpting	Sirnaomics	Two encapsulated siRNAs targeting TGF-β1 and Cox-2 to reduce fibrogenic response	Anti-fibrosis RNA interference therapeutic - Sirnaomic; Cotsiranib; STP-705; STP-705LU; STP-705LV; STP705L
Remlarsen	Phase II	NCT03601052	Completed	Pathological fibrosis, Fibrous scar & Keloid	miRagen Therapeutics (Viridian)	miR-29b mimic to reduce collagen and other scarring proteins	miR-29 replacement; MRG-201
RXI-109	Phase II	NCT02030275/NCT02246465/NCT02079168	Completed/unknown	Hypertrophic Scars & Keloids	RXi Pharmaceuticals (Phio Pharmaceuticals)	Self-delivering siRNA targeting CTGF to reduce fibrosis	RXI 109; PH-109
**Antisense Oligonucleotides (ASOs)**							
AST-005	Phase I	NCT01290692	Completed	Psoriasis	Purdue Pharma, Exicure	Nanoparticle-based spherical nucleic acid (SNA) to knockdown a tumor necrosis factor gene; Tumour necrosis factor inhibitors	AST-005
MRG 110	Phase I	NCT03603431	Completed	wound healing	Viridian Therapeutics; miRagen Therapeutics, Inc.	Inhibitor of microRNA-92	Anti Mir92a; MRG-110; S95010
XCUR17	Phase I	Unknown	Completed	Alopecia; Netherton syndrome; Psoriasis	Dermelix Biotherapeutics; Exicure	SNA targeted to mRNA encoding interleukin 17 receptor alpha (IL-17RA)	XCUR17
QR 313	Phase I/II	NCT05529134	Active	Epidermolysis bullosa	Phoenicis Therapeutics; ProQR Therapeutics	Designed to exclude exon 73 from the mRNA (exon skipping) and produce a functional C7 protein, thereby restoring the functionality of the anchoring fibrils	PTW-002; QR-313; QRX-313
QR 313	Phase I/II	NCT03605069	Terminated	Fibrous scar & Keloid	Phoenicis Therapeutics	Designed to exclude exon 73 from the mRNA (exon skipping) and produce a functional C7 protein, thereby restoring the functionality of the anchoring fibrils	PTW-002; QR-313; QRX-313
SB011	Phase II	NCT02079688	Completed	Atopic dermatitis	Sterna Biologicals GmbH & Co. KG	DNAzyme hgd40 targeting GATA3, a highly mutated transcription factor	hgd40; SB-010; SB-011; SB-012
Cobomarsen	Phase II	NCT03837457	Terminated	Cutaneous T-Cell Lymphoma; Dystropic Epidemolysis Bulosa	Viridian Therapeutics	Inhibitor of miR-155	MRG-106
PF-06473871	Phase II	NCT02205476	Terminated	Hypertrophic scar	Pfizer	Anti-CTGF antisense oligonucleotide	EXC-001; PF-06473871; PF-6473871
Trabedersen	Phase II/III	NCT00844064	Completed	Melanoma	Oncotelic Therapeutics	mRNA of the human TGF-β2 gene	AP 2/09-DS; AP-12009; AP-2/09; OT-101; OT-201; Personalised dosing TGF-beta antisense; TGF-beta antisense
Donidalorsen	Phase III	NCT05392114	Active	Hereditary angioedema	Ionis Pharma	Reduce the production of prekallikrein	Donidalorsen sodium; IONIS-PKK-L Rx; ISIS 721744
Tilsotolimod	Phase III	NCT04126876	Active	Malignant melanoma; Anti-programmed cell refractory death protein 1 (PD-1) metastatic melanoma	Idera/Bristol Myers Squibb	Immunologic cytotoxicity; Toll-like receptor 9 agonists	IMO-2125; IMO-2125 sodium; Tilsotolimod sodium
**Messenger RNA (mRNA)**							
Lipo-MERIT	Phase I	NCT02410733	Active	Advanced Melanoma	BioNTech SE	mRNA encoding TAAs: NY-ESO-1, MAGE-A3, tyrosinase, and TPTE	None
BNT131	Phase I	NCT03871348	Active	Malignant melanoma	BioNTech/Sanofi	mRNA encoding cytokines: IL-12sc, IL-15sushi, IFNa and GM-CSF; BioNTech FixVac platform	BNT-131; SAR-441000
mRNA-2752	Phase I	NCT03739931	Active	Immune Checkpoint Refractory Melanoma, Relapsed/Refractory Solid Tumor Malignancies	Moderna Therapeutics (Collaborator: AstraZeneca)	mRNA encoding OX40L T cell co-stimulator, IL-23, and IL-36γ pro-inflammatory cytokines; immunostimulant	iTu triple combination - Moderna Therapeutics; mRNA intratumoral immuno-oncology therapeutics - Moderna Therapeutics; mRNA 2752; OX40L/IL-23/IL-36γ
JCXH-211	Phase I	NCT05539157	Active	Malignant melanoma	Immorna Biotherapeutics, Inc.	self-replicating mRNA encoding cytokine IL-12	JCXH 211
RBL001/RBL002	Phase I	NCT01684241	Completed	Melanoma	BioNTech RNA Pharmaceuticals GmbH (BioNTech SE)	mRNA encoding TAAs: RBL001/RBL002	MERIT; RB0001; RB_0001; RBL-001/RBL-002; RBL-002/RBL-001
BNT121	Phase I	NCT02035956	Completed	Malignant Melanoma, Unresectable Malignant Melanoma stage IIIA-C and IV	BioNTech RNA Pharmaceuticals GmbH (BioNTech SE)	Poly-neo-epitopic encoding mRNAs specific to patient mutanome with and without the RBL001/RBL002 antigen-encoded mRNA	BNT-121; IVAC MUTANOME; Melanoma RNA vaccine personalised - BioNTech/Ribological/TRON; Personalised melanoma vaccine - BioNTech/TRON
NEO-PV-01	Phase I	NCT02897765	Completed	Malignant Melanoma	BioNTech US Inc. (Collaborator: Bristol-Myers Squibb)	mRNA encoding neoantigens for a personalized cancer vaccine to patient mutanome; immunoglobulin stimulant	Neo Vax; NEO-PV-01; Neoantigen vaccine; Neoantigen-based vaccine - BioNTech; NeoAntigen-peptides; Personalized-neoantigen-cancer-vaccine-BioNTech; Personalized-neoantigen-vaccine-BioNTech
NEO-PV-01	Phase I	NCT03597282	Terminated	Metastatic Melanoma	BioNTech US Inc. (Collaborator: Apexigen, Inc.)	mRNA encoding neoantigens for a personalized cancer vaccine to patient mutanome; immunoglobulin stimulant	None
Poly-ICLC	Phase I	NCT01970358	Completed	Melanoma	Patrick Ott, Md, Dana-Farber Cancer Institute; Oncovir, Inc.	mRNA encoding neoantigens (up to 20 TAAs) for a personalized cancer vaccine to patient mutanome	NeoVax; Hiltonol
ECI-006	Phase I	NCT03394937	Terminated	Melanoma	eTheRNA immunotherapies	mRNA encoding TAAs: tyrosinase, GP100, MAGE-A3, MAGE-C2, and PRAME. Administered with mRNA encoding immunostimulants (TriMix)	ECI 006
BNT122	Phase I/II	NCT03815058/NCT03289962	Active	Untreated Advanced Melanoma	Genentech, Inc. (Collaborator: BioNTech SE)	mRNA encoding neoantigens for a personalized cancer vaccine to patient mutanome; BioNTech iNest platform	BNT-122; BNT122/RO7198457; Autogene cevumeran; Individualized Neoantigen Specific immunotherapy; iNeST; IVAC_M_uID; PCV RO7198457; Personalized cancer vaccine RO7198457; RG-6180; RG6180-1; RO-7198457
GM-CSF	Phase I/II	NCT00204516	Completed	Malignant Melanoma	University Hospital Tuebingen (Collaborator: German Research Foundation)	mRNA encoding TAAs: melan-A, MAGE-A1, MAGE-A3, survivin, GP100, and tyrosinase	Molgramostim; CSF 39300; GMC 89107; Leucomax; SCH 39300
National Cancer Institute (NCI)-4650	Phase I/II	NCT03480152	Terminated	Melanoma	National Cancer Institute (NCI); Moderna Therapeutics	mRNA encoding neoantigens (up to 15 TAAs) for a personalized cancer vaccine against those expressed by autologous tumor cells	mRNA 4650; NCI 4650; mRNA-based PCV NCI-4650; NC-I4650
BNT111	Phase II	NCT04526899	Active	Melanoma Stage III, Melanoma Stage IV, Unresectable Melanoma, Anti-PD-1-refractory/relapsed melanoma, malignant melanoma	BioNTech SE (Collaborator: Regeneron Pharmaceuticals)	mRNA encoding TAAs: NY-ESO-1, MAGE-A3, tyrpsinase, TPTE; BioNTech FixVac platform	BNT-111; Lipo-MERIT; RB 0003; RB_0003; RBL 001/RBL 002/RBL 003/RBL 004; RBL001.1/RBL002.2/RBL003.1/RBL004.1; RNA-LPX; RNA(LIP); Tetravalent RNA-lipoplex Cancer Vaccine
mRNA-4157/V940	Phase II	NCT03897881	Active	Malignant Melanoma	Moderna Therapeutics. (Collaborator: Merck Sharp & Dohme LLC)	mRNA encoding neoantigens for a personalized cancer vaccine to patient mutanome; immunostimulant	mRNA 4157; PCV; Personalized Cancer Vaccine - Moderna Therapeutics
GSK1572932A	Phase II	NCT00849875	Terminated	Melanoma	GlaxoSmithKline	mRNA encoding single TAA: MAGE-A3	Zastumotide; Astuprotimut-R; D1/3 MAGE-3 fusion protein; D1/3 MAGE-3 fusion protein SB MAGE-3; D1/3 MAGE-3 His; D1/3 MAGE-3 His fusion protein; GSK 1572932A; GSK2132231A; GSK 249553; GSK1203486A; MAGE-A3; MAGE-A3 antigen specific cancer immunotherapeutic; MAGE-A3 ASCI; NSC 719274; SB 249553; SB MAGE-3; SID 534984
**Other RNAs**							
CV 8102	Phase I	NCT03291002	Active	Melanoma, Squamous Cell Carcinoma of the Skin	CureVac AG (Collaborators: Syneos health and Cromos Pharma LLC)	Non-coding, non-capped ssRNA (CV8102) complexed with a cationic peptide to induce an immune response via TLR-7/8 and DDX58/RIG-1; immunostimulant	CV8102
BO 112	Phase II	NCT04570332	Active	Malignant melanoma	Highlight therapeutics	non-coding double stranded synthetic RNA activating TLR3, RIG-1, and MDA5 to sensitize tumor cells to immune response	BO-112; Nanoplexed Poly IC BO-112; Nanoplexed Polyinosinic:Polycytidylic Acid BO-112

## References

[1] Damase TR, The Limitless Future of RNA Therapeutics. Front Bioeng Biotechnol 9, 628137, doi: 10.3389/fbioe.2021.628137 (2021). 33816449 PMC8012680

[2] Kalal BS, Upadhya D, Pai VR. Chemotherapy Resistance Mechanisms in Advanced Skin Cancer. Oncol Rev 11, 326, doi:10.4081/oncol.2017.326 (2017). 28382191 PMC5379221

[3] Li X Targeting microRNA for improved skin health. Health Sci Rep 4, e374, doi: 10.1002/hsr2.374 (2021). 34667882 PMC8506131

[4] Banerjee J, Chan YC, Sen CK. MicroRNAs in skin and wound healing. Physiol Genomics 43, 543–556, doi: 10.1152/physiolgenomics.00157.2010 (2011). 20959495 PMC3110888

[5] Bartel DP. Metazoan MicroRNAs. Cell 173, 20–51, doi: 10.1016/j.cell.2018.03.006 (2018). 29570994 PMC6091663

[6] Hu B Therapeutic siRNA: state of the art. Signal Transduction and Targeted Therapy 5, 101, doi: 10.1038/s41392-020-0207-x (2020). 32561705 PMC7305320

[7] Lam JK, Chow MY, Zhang Y, siRNA Versus miRNA as Therapeutics for Gene Silencing. Mol Ther Nucleic Acids 4, e252, doi: 10.1038/mtna.2015.23 (2015). 26372022 PMC4877448

[8] Zhu Y, Zhu L, Wang X, RNA-based therapeutics: an overview and prospectus. Cell Death Dis 13, 644, doi: 10.1038/s41419-022-05075-2 (2022). 35871216 PMC9308039

[9] Bejar N, Tat TT, Kiss DL. RNA Therapeutics: the Next Generation of Drugs for Cardiovascular Diseases. Curr Atheroscler Rep 24, 307–321, doi:10.1007/s11883-022-01007-9 (2022). 35364795 PMC8975710

[10] Makino K Anti-connective tissue growth factor (CTGF/CCN2) monoclonal antibody attenuates skin fibrosis in mice models of systemic sclerosis. Arthritis Res Ther 19, 134, doi: 10.1186/s13075-017-1356-3 (2017). 28610597 PMC5470189

[11] Kang S RNAi nanotherapy for fibrosis: highly durable knockdown of CTGF/CCN-2 using siRNA-DegradaBALL (LEM-S401) to treat skin fibrotic diseases. Nanoscale 12, 6385–6393, doi: 10.1039/c9nr10305h (2020). 32134425

[12] Carswell L, Borger J. Hypertrophic Scarring Keloids, <https://www.ncbi.nlm.nih.gov/pubmed/30725743> (2022). 30725743

[13] Roberts TC, Langer R, Wood MJA. Advances in oligonucleotide drug delivery. Nat Rev Drug Discov 19, 673–694, doi: 10.1038/s41573-020-0075-7 (2020). 32782413 PMC7419031

[14] Crooke ST, Baker BF, Crooke RM, Antisense technology: an overview and prospectus. Nat Rev Drug Discov 20, 427–453, doi: 10.1038/s41573-021-00162-z (2021). 33762737

[15] Quemener AM, The powerful world of antisense oligonucleotides: From bench to bedside. Wiley Interdiscip Rev RNA 11, e1594, doi: 10.1002/wrna.1594 (2020). 32233021 PMC9285911

[16] Di Fusco D Antisense Oligonucleotide: Basic Concepts and Therapeutic Application in Inflammatory Bowel Disease. Front Pharmacol 10, 305, doi: 10.3389/fphar.2019.00305 (2019). 30983999 PMC6450224

[17] Reese CB. The chemical synthesis of oligo-and poly-nucleotides by the phosphotriester approach. Tetrahedron 34, 3143–3179 (1978).

[18] Orr RM. Technology evaluation: fomivirsen, Isis Pharmaceuticals Inc/CIBA vision. Curr Opin Mol Ther 3, 288–294 (2001). 11497353

[19] Chan JH, Lim S, Wong WS. Antisense oligonucleotides: from design to therapeutic application. Clin Exp Pharmacol Physiol 33, 533–540, doi: 10.1111/j.1440-1681.2006.04403.x (2006). 16700890

[20] Juliano RL, Carver K. Cellular uptake and intracellular trafficking of oligonucleotides. Adv Drug Deliv Rev 87, 35–45, doi: 10.1016/j.addr.2015.04.005 (2015). 25881722 PMC4504789

[21] Liang XH, Sun H, Nichols JG. RNase H1-Dependent Antisense Oligonucleotides Are Robustly Active in Directing RNA Cleavage in Both the Cytoplasm and the Nucleus. Mol Ther 25, 2075–2092, doi: 10.1016/j.ymthe.2017.06.002 (2017). 28663102 PMC5589097

[22] Desterro J, Bak-Gordon P, Carmo-Fonseca M. Targeting mRNA processing as an anticancer strategy. Nat Rev Drug Discov 19, 112–129, doi: 10.1038/s41573-019-0042-3 (2020). 31554928

[23] Juliano RL. The delivery of therapeutic oligonucleotides. Nucleic Acids Res 44, 6518–6548, doi: 10.1093/nar/gkw236 (2016). 27084936 PMC5001581

[24] Younis HS, in A Comprehensive Guide to Toxicology in Preclinical Drug Development (ed Faqi Ali S.) Ch. 26, 647–664 (Academic Press, 2013).

[25] Wan J, Bauman JA, Graziewicz MA, Oligonucleotide therapeutics in cancer. Cancer Treat Res 158, 213–233, doi: 10.1007/978-3-642-31659-3_9 (2013). 24222360

[26] Agrawal S Mixed-backbone oligonucleotides as second generation antisense oligonucleotides: in vitro and in vivo studies. Proc Natl Acad Sci U S A 94, 2620–2625, doi: 10.1073/pnas.94.6.2620 (1997). 9122245 PMC20138

[27] Sardone V, Zhou H, Muntoni F, Antisense Oligonucleotide-Based Therapy for Neuromuscular Disease. Molecules 22, doi: 10.3390/molecules22040563 (2017). PMC6154734 28379182

[28] Wahlestedt C, Potent and nontoxic antisense oligonucleotides containing locked nucleic acids. Proc Natl Acad Sci U S A 97, 5633–5638, doi: 10.1073/pnas.97.10.5633 (2000). 10805816 PMC25880

[29] Havens MA, Hastings ML. Splice-switching antisense oligonucleotides as therapeutic drugs. Nucleic Acids Res 44, 6549–6563, doi: 10.1093/nar/gkw533 (2016). 27288447 PMC5001604

[30] Gallant-Behm CL, A synthetic microRNA-92a inhibitor (MRG-110) accelerates angiogenesis and wound healing in diabetic and nondiabetic wounds. Wound Repair Regen 26, 311–323, doi: 10.1111/wrr.12660 (2018). 30118158

[31] Huang CK, Kafert-Kasting S, Thum T. Preclinical and Clinical Development of Noncoding RNA Therapeutics for Cardiovascular Disease. Circ Res 126, 663–678, doi: 10.1161/CIRCRESAHA.119.315856 (2020). 32105576 PMC7043728

[32] Wolff JA, Direct gene transfer into mouse muscle in vivo. Science 247, 1465–1468, doi: 10.1126/science.1690918 (1990). 1690918

[33] Weide B, Results of the first phase I/II clinical vaccination trial with direct injection of mRNA. J Immunother 31, 180–188, doi: 10.1097/CJI.0b013e31815ce501 (2008). 18481387

[34] Gu Y, Duan J, Yang N, mRNA vaccines in the prevention and treatment of diseases. MedComm (2020) 3, e167, doi: 10.1002/mco2.167 (2022). 36033422 PMC9409637

[35] van Dulmen M, Rentmeister A. mRNA Therapies: New Hope in the Fight against Melanoma. Biochemistry 59, 1650–1655, doi: 10.1021/acs.biochem.0c00181 (2020). 32298088

[36] ClinicalTrials.gov. <https://clinicaltrials.gov/ct2/show/NCT02410733?cond=NCT02410733&draw=2> (2022a).

[37] Sahin U, An RNA vaccine drives immunity in checkpoint-inhibitor-treated melanoma. Nature 585, 107–112, doi:10.1038/s41586-020-2537-9 (2020). 32728218

[38] ClinicalTrials.gov. <https://clinicaltrials.gov/> (2022b).

[39] AdisInsight. <https://adisinsight.springer.com/> (2023).

[40] BioNTech. <https://investors.biontech.de/news-releases/news-release-details/biontech-receives-fda-fast-track-designation-its-fixvac> (2021).

[41] BioNTech. <https://www.biontech.com/int/en/home/pipeline-and-products/platforms/our-mrna-platforms.html> (2023).

[42] clinicaltrials.gov. <https://clinicaltrials.gov/ct2/show/NCT04526899 > (2022c)

[43] Moderna. <https://investors.modernatx.com/news/news-details/2022/Moderna-and-Merck-Announce-mRNA-4157V940-an-Investigational-Personalized-mRNA-Cancer-Vaccine-in-Combination-with-KEYTRUDAR-pembrolizumab-Met-Primary-Efficacy-Endpoint-in-Phase-2b-KEYNOTE-942-Trial/default.aspx#> (2022).

[44] Vitale G Chemical & Engineering News, <https://cen.acs.org/pharmaceuticals/vaccines/ModernaMerck-cancer-vaccine-shows-promise/100/web/2022/12?utm_source=LatestNews&utm_medium=LatestNews&utm_campaign=CENRSS> (2023).

